# Transfer Learning for Risk Classification of Social Media Posts: Model Evaluation Study

**DOI:** 10.2196/15371

**Published:** 2020-05-13

**Authors:** Derek Howard, Marta M Maslej, Justin Lee, Jacob Ritchie, Geoffrey Woollard, Leon French

**Affiliations:** 1 Campbell Family Mental Health Research Institute Centre for Addiction and Mental Health Toronto, ON Canada; 2 Krembil Centre for Neuroinformatics Centre for Addiction and Mental Health Toronto, ON Canada; 3 Department of Biochemistry University of Toronto Toronto, ON Canada; 4 Department of Computer Science University of Toronto Toronto, ON Canada; 5 Department of Medical Biophysics University of Toronto Toronto, ON Canada; 6 Princess Margaret Cancer Centre University Health Network Toronto, ON Canada; 7 Institute for Medical Science University of Toronto Toronto, ON Canada; 8 Division of Brain and Therapeutics Department of Psychiatry University of Toronto Toronto, ON Canada

**Keywords:** triage, classification, natural language processing, transfer learning, machine learning, data interpretation, statistical, mental health, social support

## Abstract

**Background:**

Mental illness affects a significant portion of the worldwide population. Online mental health forums can provide a supportive environment for those afflicted and also generate a large amount of data that can be mined to predict mental health states using machine learning methods.

**Objective:**

This study aimed to benchmark multiple methods of text feature representation for social media posts and compare their downstream use with automated machine learning (AutoML) tools. We tested on datasets that contain posts labeled for perceived suicide risk or moderator attention in the context of self-harm. Specifically, we assessed the ability of the methods to prioritize posts that a moderator would identify for immediate response.

**Methods:**

We used 1588 labeled posts from the Computational Linguistics and Clinical Psychology (CLPsych) 2017 shared task collected from the Reachout.com forum. Posts were represented using lexicon-based tools, including Valence Aware Dictionary and sEntiment Reasoner, Empath, and Linguistic Inquiry and Word Count, and also using pretrained artificial neural network models, including DeepMoji, Universal Sentence Encoder, and Generative Pretrained Transformer-1 (GPT-1). We used Tree-based Optimization Tool and Auto-Sklearn as AutoML tools to generate classifiers to triage the posts.

**Results:**

The top-performing system used features derived from the GPT-1 model, which was fine-tuned on over 150,000 unlabeled posts from Reachout.com. Our top system had a macroaveraged F1 score of 0.572, providing a new state-of-the-art result on the CLPsych 2017 task. This was achieved without additional information from metadata or preceding posts. Error analyses revealed that this top system often misses expressions of hopelessness. In addition, we have presented visualizations that aid in the understanding of the learned classifiers.

**Conclusions:**

In this study, we found that transfer learning is an effective strategy for predicting risk with relatively little labeled data and noted that fine-tuning of pretrained language models provides further gains when large amounts of unlabeled text are available.

## Introduction

Mental health disorders are highly prevalent, with epidemiological studies reporting roughly half the population in the United States meeting the criteria for one or more mental disorders in their lifetime and roughly a quarter meeting the criteria in a given year [1]. Available survey evidence suggests that the first onset of mental health disorders is typically in childhood or adolescence and that later-onset disorders are mostly secondary conditions. The severity of mental disorders is highly related to their comorbidity, with complex interactions among disorders [2]. Moreover, severe disorders tend to be preceded by less severe disorders that are often not brought to clinical attention, indicating a need for early detection and intervention strategies [3,4].

Mental disorders are among the strongest predictors for nonsuicidal self-injury and suicidal behaviors; however, little is known about how people transition from suicidal thoughts to attempts [5]. Given the high incidence of mental health disorders and the relatively low incidence of suicide attempts, predicting the risk for suicidal behavior is difficult. In particular, Franklin et al [6] report a lack of progress over the last 50 years on the identification of risk factors that can aid in the prediction of suicidal thoughts and behaviors. However, they also proposed that new methods with a focus on risk algorithms using machine learning present an ideal path forward. These approaches can be integrated into peer support forums to develop repeated and continuous measurements of a user’s well-being to inform early interventions.

Peer support forums can be a useful and scalable approach to social therapy for mental health issues [7]. Many individuals are already seeking health information online, and this manner of information access can help those who are reluctant to seek professional help, are concerned about stigma or confidentiality, or face barriers to access [8]. There is limited evidence showing that online peer support without professional moderation is an effective strategy for enhancing users’ well-being [7,9]. However, in a systematic review of social networking sites for mental health interventions, Ridout and Campbell [10] identified the use of moderators as a key component of successful interventions on these Web-based platforms. The development of automated triage systems in these contexts can facilitate professional intervention by prioritizing users for specialized care [11,12] or decreasing response time when a risk for self-harm is identified [13]. Although the computational infrastructure of peer support forums is scalable, the effectiveness of human moderation is challenging to grow with community size. If they are accurate, automated systems can address these needs through computational approaches that are fast and scalable.

Previous research suggests that the language of individuals with mental health conditions is characterized by distinct features [14-17], eg, frequent use of first-person singular pronouns has been associated with depression [18]. This has sparked efforts to develop automated systems that, when given social media data, can predict the same level of suicide or self-harm risk that a trained expert would predict.

Such automated systems typically start with a feature extraction step that converts the variable length input text into fixed-length numeric vectors (features). This step is required to apply machine learning classifiers that operate on such vectors. An example is the bag-of-words representation, where each numeric feature represents the count of a specific word that is selected based on frequency or from a lexicon. With such a representation, a classifier may learn that mentions of *hopeless* are more common in text written by depressed individuals. This step of extracting features that best represent the text is a key part of such systems because a significant amount of information loss can occur. For example, in the bag-of-words representation, the order of the words is discarded. In contrast, differences in performance across machine learning classifiers are lower when representations are held constant. For example, good classifiers will have a similar performance on the same representations. Lexicon-based tools are highly dependent on their dictionaries, which require manual curation and validation. However, lexicon- and rule-based approaches are typically more interpretable than more complex neural network–based representations.

Recently, word embeddings have been shown to provide rich representations where words from the same context of a corpus tend to occupy a similar feature space [19]. The use of these embeddings has significantly boosted performance in several natural language processing tasks in recent years [20]. Generating such word embeddings can be done by building a neural network model that predicts a word given its neighboring words or vice versa. These word representations are learned from large corpora. These representations can be reused for other tasks. For example, a pretrained representation of *hopeless* would be similar to *despair*, allowing a classifier to group text that shares these words. This reuse is a type of transfer learning, which allows for the knowledge learned from one domain to be transferred to a task in an adjacent domain [21]. More recently, pretrained word representations have been shown to capture complex contextual word characteristics better than the preceding shallow models [22]. The fine-tuning of large pretrained language models in an unsupervised fashion has pushed forward the applicability of these approaches in cases with small amounts of labeled data [20,23]. Such fine-tuning could alter the learned context of *worries* to account for its placement in the common Australian expression of *no worries* when being transferred from an American to Australian corpus. Given these recent advances in natural language processing, we tested the performance of transfer learning with pretrained language models on risk classification of social media posts.

Reachout.com is an Australian youth-based mental health peer support forum. It is targeted for those aged 14 to 25 years, and the community is maintained by staff and trained volunteer moderators. Staff and moderators monitor the forums, and they respond, as required, with empathy, support, and referrals to relevant information and available services. The 2017 Computational Linguistics and Clinical Psychology (CLPsych)–shared task organizers provided a corpus of posts from Reachout.com to assess the ability of automated methods to triage forum posts based on the urgency of moderator response [24]. For example, posts that suggest the author might hurt themselves or others are labeled as being high in priority for moderator response (*crisis*). We noted that these labels do not distinguish if the author is contemplating self-harm, nonsuicidal self-injury, or suicide. These constructs have different prevalence and incidence rates [25]. This dataset is small and imbalanced as the majority of posts are labeled as not requiring a moderator response. For example, only 5.2% (82/1588) of the posts are labeled as *crisis*. Given the higher importance of posts requiring moderator response, the organizers of the CLPsych-shared task chose the macroaveraged F1 metric to weight performance equally across the labels that mark the urgency of moderator response. This metric weighs each of those labels for both precision and recall equally. As a result, misclassification of a *crisis* post will be costlier because *crisis* posts occur less frequently. Several advanced methods have been applied to this dataset [24,26], but a systematic evaluation of feature extraction methods has not been performed.

In this paper, we benchmarked multiple feature extraction methods on forum posts from Reachout.com by evaluating their ability to predict the urgency of moderator response. Furthermore, we explored the interpretability through emoji representations and by visualizing word importance in text that mimics themes from suicide notes. We have shown that modern transfer learning approaches that take advantage of large corpora of unlabeled text, in combination with automated machine learning (AutoML) tools, improve performance.

## Methods

### Data

#### Reachout.com

Our primary data source was made available for the 2017 CLPsych-shared task and was collected from the Australian mental health peer support forum, Reachout.com [13,24]. The entire dataset consisted of 157,963 posts written between July 2012 and March 2017. Of those, 1188 were labeled and used for training the classification system, and 400 labeled posts were held out for the final evaluation of the systems. Posts were labeled *green* (58.6%, 931/1588), *amber* (25.6%, 390/1588), *red* (11.7%, 185/1588), or *crisis* (5.2%, 82/1588) based on the level of urgency with which moderators should respond. The postannotation task began with the 3 judges (organizers of the shared task) discussing and coming to a shared agreement on the labels for roughly 200 posts, guided by informal annotation and triage criteria provided by Reachout. The annotators ultimately formalized their process in a flowchart to standardize the labeling process and included fine-grained or granular annotations for each of the posts (Summary table of fine-grained labels in Multimedia Appendix 1). They then annotated the remaining posts independently, and the interannotator agreement was measured over these posts (excluding 22 posts labeled ambiguous by at least one judge). The 3 judges achieved a Fleiss kappa of 0.706 and a pairwise Cohen kappa score ranging from 0.674 to 0.761, which is interpreted as substantial agreement by Viera and Garrett [27]. The above mentioned steps, evaluations, and development of this dataset were previously undertaken by Milne et al [13,24].

#### University of Maryland Reddit Suicidality Dataset

To test the generalizability of the system developed on the Reachout.com data, we used a subset of the data made available from the University of Maryland (UMD) Reddit Suicidality Dataset [28,29]. The collection of this dataset followed an approach where the initial signal for a positive status of suicidality was a user having posted in the subreddit, /r/SuicideWatch, between 2006 and 2015. Annotations were then applied at the user level based on their history of posts. We used the subset that was curated by expert annotators to assess suicide risk. These volunteer experts include a suicide prevention coordinator for the Veteran’s Administration; a cochair of the National Suicide Prevention Lifelines Standards, Training, and Practices Subcommittee; a doctoral student with expert training in suicide assessment and treatment whose research is focused on suicidality among minority youths; and a clinician in the Department of Emergency Psychiatry at Boston Children’s Hospital. Two sets of annotator instructions (short and long) were used, following an adapted categorization of suicide risk developed by Corbitt-Hall et al [30]: (a) no risk (or *None*): I don’t see evidence that this person is at risk for suicide, (b) low risk: There may be some factors here that could suggest risk, but I don’t really think this person is at much of a risk of suicide, (c) moderate risk: I see indications that there could be a genuine risk of this person making a suicide attempt, and (d) severe risk: I believe this person is at high risk of attempting suicide in the near future. These categories correspond roughly to the green, amber, red, and crisis categories defined in the Reachout.com data. The longer set of annotation instructions also identified 4 families of risk factors (ie, thoughts, feelings, logistics, and context). A pairwise Krippendorff alpha was used to assess interannotator agreement, with an average alpha of .812 satisfying the recommendation of a reliability cutoff of alpha >.800 [31]. Consensus labels were determined using a model for inferring true labels from multiple noisy annotations [32,33]. The abovementioned steps and development of this dataset were undertaken by Shing and et al [29].

Of the subset with labels by expert annotators, we then selected only data from users who had posted once in /r/SuicideWatch to minimize ambiguity in understanding which of their posts was the cause of the associated label. Predictions were made only on posts from /r/SuicideWatch. In total, there were 179 user posts across the categories (*a*: 32, *b*: 36, *c*: 85, and *d*: 26). The Centre for Addiction and Mental Health Research Ethics Board approved the use of this dataset for this study.

To better gauge our performance on the UMD Reddit Suicidality Dataset posts, we calculated an empirical distribution of random baselines for the macro-F1 metric. This baseline distribution quantifies the performance of random shuffles of the true labels (including the class *a* or *no risk* labels). As expected, across 10,000 of these randomizations, the mean macro-F1 was 0.25. We set a threshold of 0.336, which is 62 of 10,000 random runs to mark Reddit validation performance as better than chance (1/20 × 1/8 × 10000), corresponding to *P*<.05 and a Bonferroni correction for 8 tests (number of feature sets tested).

### Composite Quotes

We used 10 composite quotes to share example predictions of our system on text that could be predictive/indicative of self-harming and/or suicidality. These composite quotes were created by Furqan et al [34] and were derived from qualitative research that synthesized primary themes noted in a selection of suicide notes that made explicit mentions of mental illness or mental health care. To assess the role of individual words (or tokens) in the classification of the quote, we iteratively perturbed each token and replaced it with an unknown token outside of the model’s vocabulary and reran the prediction.

### Data Preprocessing and Feature Extraction

Features were extracted from only the text body of the posts. For all posts, any quotes from previous posts or links to images were removed.

We extracted features using lexicon-based tools such as Valence Aware Dictionary and sEntiment Reasoner (VADER; 4 features) [35], Linguistic Inquiry and Word Count (LIWC; 70 features) [36], and Empath (195 features) [37], which have proven to be useful for characterizing social media text and extracting psychologically relevant signals. Features were also extracted from 3 pretrained artificial neural network models: DeepMoji [38] was used to extract sentiment- and emotion-related features (eg, the use of emoticons in social media text), the Universal Sentence Encoder version 2 (using a deep averaging network encoder) (Google) [39] obtained from Tensorflow Hub that was specifically designed to facilitate transfer learning, and the Generative Pretrained Transformer (GPT) network version 1 (OpenAI) [20]. For DeepMoji, we extracted features that represent the 64 predicted emojis and the neural activations from the preceding attention layer in the network (2304 features, referred to as DeepMoji). We used the Indico Data Solutions implementation to extract features from the default pretrained GPT-1 network and also after fine-tuning on the unlabeled corpus of posts from Reachout.com [40]. All language model fine-tuning was done with 3 epochs over the unlabeled posts, as suggested by the GPT-1 authors.

With Empath and LIWC, sentence splitting was not performed. With the remaining feature encoding (VADER, DeepMoji, Universal Sentence Encoder, and both GPT models) methods, we first preprocessed the text body of each post into sentences using the sentence boundary detection from spaCy version 2.1. Sentence feature vectors were aggregated to the post level by taking their mean, maximum, and minimum for each extracted feature.

### Model Optimization and Selection

To train classifiers on the various feature sets, we used 2 AutoML methods that are built upon scikit-learn [41] to optimize and select optimal models. We selected these tools over others because they are open source. Other AutoML tools may have advantages such as ease of use or better performance for different dataset sizes and dimensionality [42]. In both cases, the AutoML methods were customized to maximize the Macro-F1 score (without the *green*-labeled posts). Each model was evaluated with 10-fold stratified cross-validation with five repeats inside of the training set. We trained the classifiers to predict the granular/fine-grained labels while evaluating the final output with the same macro-F1 score of the *amber*, *red,* and *crisis* categories.

We used the Tree-based Optimization Tool (TPOT) [43], which builds and selects machine learning pipelines using genetic programming. TPOT is built to generate pipelines that maximize classification accuracy while penalizing complex pipelines. Similarly, we used Auto-Sklearn to train and build classifiers using Bayesian optimization meta-learning and ensemble construction [44]. Given the high proportion of no risk labels in the datasets tested, we note that Auto-Sklearn contains a *Rebalancer* class for handling imbalanced class distributions. We primarily used default TPOT/Auto-Sklearn parameters with a population size of 200, a maximum evaluation time for a single pipeline of 5 min and total time as a stopping parameter, typically set to 2 days.

### Mantel Tests

To compute the matrix of pairwise Euclidean distances between posts for each set of features, we used SciPy’s distance matrix function [45]. This test allows quantification of the distances between posts across the various feature spaces. This is done in an unsupervised manner across the training and test posts. We used scikit-bio’s mantel function with 999 permutations to perform the Mantel test on these distance matrices.

### Emoji Visualization

To better understand the distribution of the 64 emoji features represented across the labeled posts, we aggregated the mean of an emoji feature across sentences in a post. Each of these aggregate features was then normalized to be between 0 and 1 to better compare features against each other. To obtain a measure of feature importance, we permuted each feature column and assessed the decrease in classification performance on the macro-F1 metric while using the best-performing pipeline derived from TPOT. For each emoji feature, we performed this procedure 10,000 times. Images of the emojis were obtained from EmojiOne (currently JoyPixels Inc) and converted to grayscale.

### Availability

The CLPsych 2017 and UMD Reddit Suicidality datasets are available upon request from the original sources [28,29]. The code and instructions to fine-tune, train, and test a GPT-1 model on the CLPsych 2017 dataset is available online [46].

## Results

### Classification

**Figure 1:**
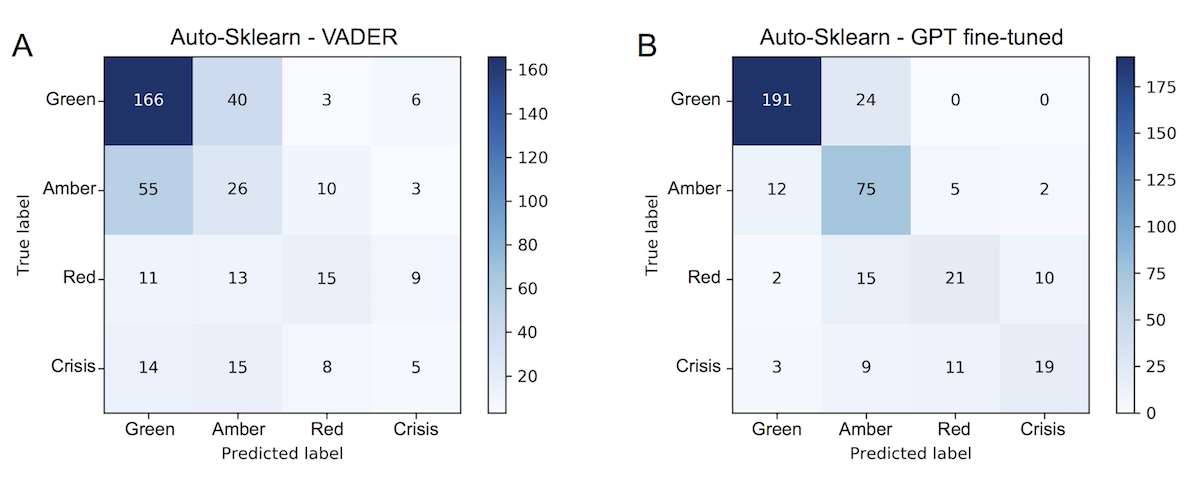
Confusion matrices for 2 models trained with Auto-Sklearn. Each cell in the matrix provides the counts of posts that were labeled in the corresponding row and column axis that represent the predicted and true labels, respectively. Counts are colored from the highest cell (blue) to the lowest (white). The top-left to bottom-right diagonal cells count correctly predicted posts. Panel A trained with Valence Aware Dictionary and sEntiment Reasoner (VADER) features. Panel B trained with features from a fine-tuned Generative Pretrained Transformer (GPT) language model.

To benchmark the performance of various text derived features for the automated classification of online forum posts, we ran both TPOT and Auto-Sklearn on the features generated from the post bodies. In Table 1, we report the average observed score across the training folds, the final score on the held-out Reachout.com test set, and the external validation performance on Reddit data of the classifier trained only on Reachout.com data. In Figure 1, we present confusion matrices from 2 separate models trained with Auto-Sklearn to better demonstrate the predictions made across the imbalanced classes. Panel A shows the predictions of the VADER features, which resulted in a macro-F1 of 0.263. Panel B shows the predictions of the top-performing system with fine-tuned GPT features (a macro-F1 of 0.572).

**Table 1:** Benchmarking by features, automated machine learning methods, and datasets with the macro-F1 metric.

Feature set	Feature count	Tree-based Optimization Tool			Auto-Sklearn		
		Train 10-fold, 5 times	Test	Reddit validation	Train 10-fold, 5 times	Test	Reddit validation
Empath (post)	195	0.280	0.253	0.385^a^	0.292	0.344	0.321
Linguistic Inquiry and Word Count	70	0.434	0.354	0.346^a^	0.433	0.380	0.315
Valence Aware Dictionary and sEntiment Reasoner (sentence)	12	0.363	0.263	0.356^a^	0.340	0.263	0.353^a^
Emoji 64	192	0.425	0.369	0.280	0.424	0.461	0.308
DeepMoji	6912	0.442	0.452	0.345^a^	0.391	0.437	0.351^a^
Universal Sentence Encoder	1536	0.457	0.446	0.300	0.484	0.479	0.236
GPT^b^ default	2304	0.373	0.334	0.344^a^	0.396	0.383	0.402^a^
GPT fine-tuned	2304	0.510	0.559	0.320	0.492	0.572	0.324

^a^Reddit validation performance better than chance.

^b^GPT: Generative Pretrained Transformer.

We noted that the average macro-F1 obtained during training was a fairly reliable predictor of the score on the held-out test set. Auto-Sklearn performed better on average than TPOT (mean test macro-F1 of 0.414 versus 0.379, respectively). We also observed the trend that features extracted from pretrained models perform better in general (average Auto-Sklearn test macro-F1 of 0.329 versus 0.466). However, the features extracted from the default GPT model (without any additional fine-tuning) were the worst performing of those obtained from neural models, whereas the GPT model that was fine-tuned on the unlabeled posts performed best across all experiments. The Universal Sentence Encoder and fine-tuned GPT features exceeded the highest macro-F1 score reached in the 2017 CLPsych-shared task when a classifier was learned with Auto-Sklearn (0.467; submission by Xianyi Xia and Dexi Liu). Upon inspection, the Auto-Sklearn–generated classifier for the GPT fine-tuned features was a complex ensemble of pipelines with multiple preprocessing steps and random forest classifiers. The TPOT-generated classification pipeline first selects features using the analysis of variance *F* value, then binarizes the values for classification with a K-nearest neighbor classifier (k=21; Euclidean distance). In contrast, the classifiers generated for the Universal Sentence Encoder features are a linear support vector machine (TPOT) and ensembles of linear discriminant analysis classifiers (Auto-Sklearn).

To better understand the low Reddit validation scores, we calculated a random baseline. Although it is random, this does use information about the class distributions. We marked Reddit validation performance as better than chance in Table 1 with an ^a^. Only classifiers learned from the VADER, DeepMoji, and default GPT features had macro-F1 scores above the threshold for both the TPOT and Auto-Sklearn learned classifiers. Unlike the CLPsych 2017 score that does not include the *green* or no risk labels, we used macro-F1 from all classes in the Reddit validation tests (corresponding to the CLPsych 2019 primary metric). When using the macro-F1 score that excluded the *no risk* class in the Reddit validation, none of the classifiers outperformed random runs at the same threshold. This is because of the classifiers having a good performance on the *no risk* or *green* labels and not the 3 remaining labels.

To better assess the variability of our best-performing system (Auto-Sklearn trained with features generated from the fine-tuned GPT model), we reran the Auto-Sklearn training and testing process 20 times. For each run, Auto-Sklearn was allotted 24 hours of compute time. Across those 20 systems, the average macro-F1 score on the held-out test set was 0.5293 (SD 0.0348). Of those 20 systems, the best- and worst-performing systems had a final test score of 0.6156 and 0.4594, respectively. Importantly, despite the variability and less compute time, the average macro-F1 score of these classifiers performed better than the scores obtained from different feature sets.

**Figure 2:**
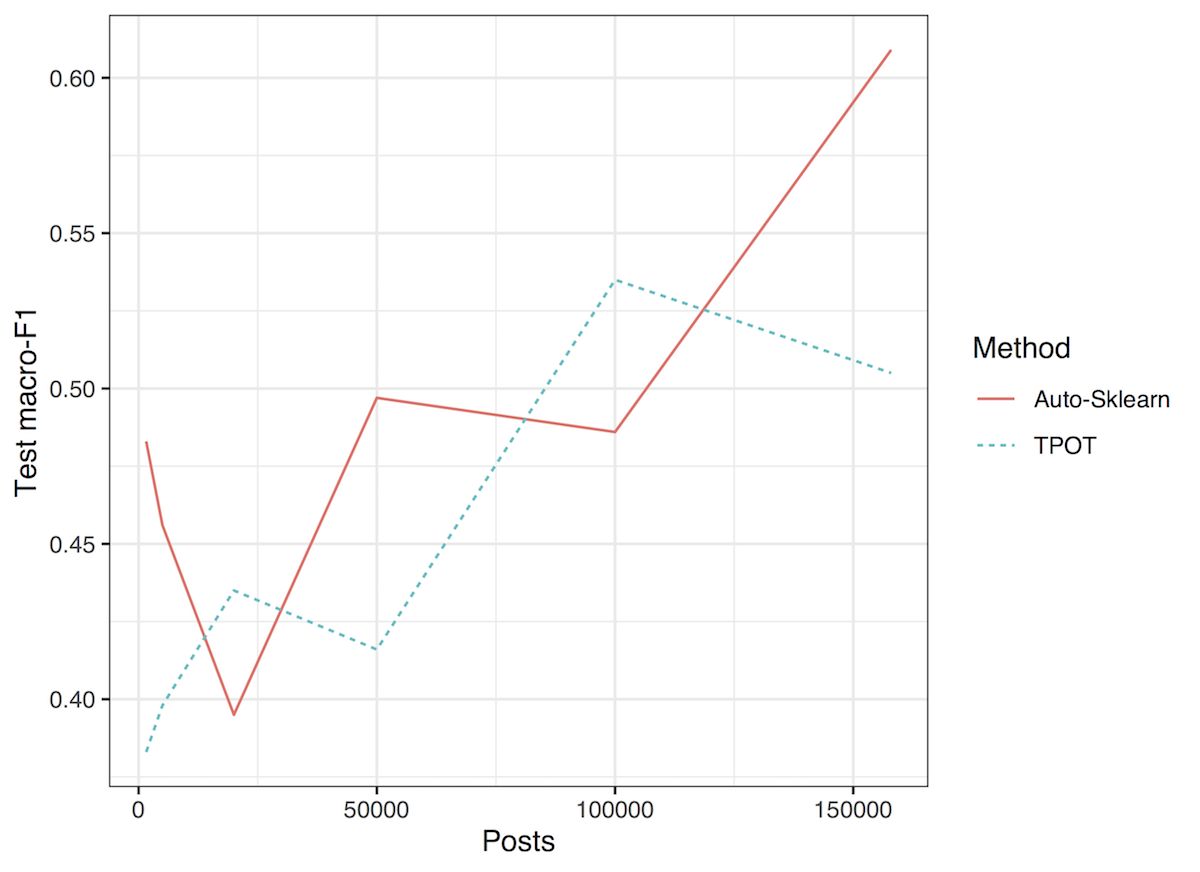
A graph of macro-F1 test scores versus the number of posts used for Generative Pretrained Transformer-1 fine-tuning. Auto-Sklearn methods are marked with continuous red (Auto-Sklearn) and dashed blue (Tree-based Optimization Tool, TPOT) lines.

To determine the impact of the amount of data used for fine-tuning the GPT model on its effectiveness for feature extraction in the classification task, we fine-tuned models with increasing amounts of unlabeled posts before extracting post-level features to train a classifier (Figure 2). Although there is significant variability, there is a general trend of better performance when using models trained on a larger amount of unlabeled data.

To compare the different representations or embeddings of the post contents, we used the Mantel test (Table 2). This compares the representations independently of their triage performance and suggests possible combinations for meta-classifiers. This test correlates the pairwise distances between posts in the benchmarked feature spaces, where a high correlation value between compared matrices indicates a significant overlap in the information they contain. Specifically, the Mantel test values range from −1 (perfect negative correlation) to 1 (perfect positive correlation), with zero representing no association between the pairs of posts in the feature spaces. Intriguingly, we observed the highest correlation between the Universal Sentence encoded features with those encoded by GPT. This is despite the comparison of aggregated DeepMoji encoded features with aggregated 64-dimensional emoji encoding of DeepMoji, which we expected to have the strongest relationship. Similarly, comparisons between the default GPT and the fine-tuned version were slightly lower than correlations with the Universal Sentence Encoder. Although it is unclear, we presumed some of these differences may be due to the aggregation of sentence-level features into a post-level representation. None of the correlations with Empath features were significant, which probably reflects the sparsity of these features.

**Table 2:** Mantel correlations between the extracted feature sets.

Feature Set	VADER^a^	Empath	LIWC^b^	Universal Sentence	Emoji 64	DeepMoji	GPT^c^ default	GPT fine-tuned
VADER	1.000	0.003	0.098	0.453	0.211	0.422	0.430	0.429
Empath	0.003	1.000	0.009	0.006	−0.005	−0.008	0.004	0.001
LIWC	0.098	0.009	1.000	0.148	0.403	0.507	0.267	0.253
Universal Sentence	0.453	0.006	0.148	1.000	0.193	0.509	0.823	0.823
Emoji 64	0.211	−0.005	0.403	0.193	1.000	0.523	0.302	0.335
DeepMoji	0.422	−0.008	0.507	0.509	0.523	1.000	0.632	0.631
GPT default	0.430	0.004	0.267	0.823	0.302	0.632	1.000	0.799
GPT fine-tuned	0.429	0.001	0.253	0.823	0.335	0.631	0.799	1.000

^a^VADER: Valence Aware Dictionary and sEntiment Reasoner.

^b^LIWC: Linguistic Inquiry and Word Count.

^c^GPT: Generative Pretrained Transformer.

### System Interpretability

**Figure 3:**
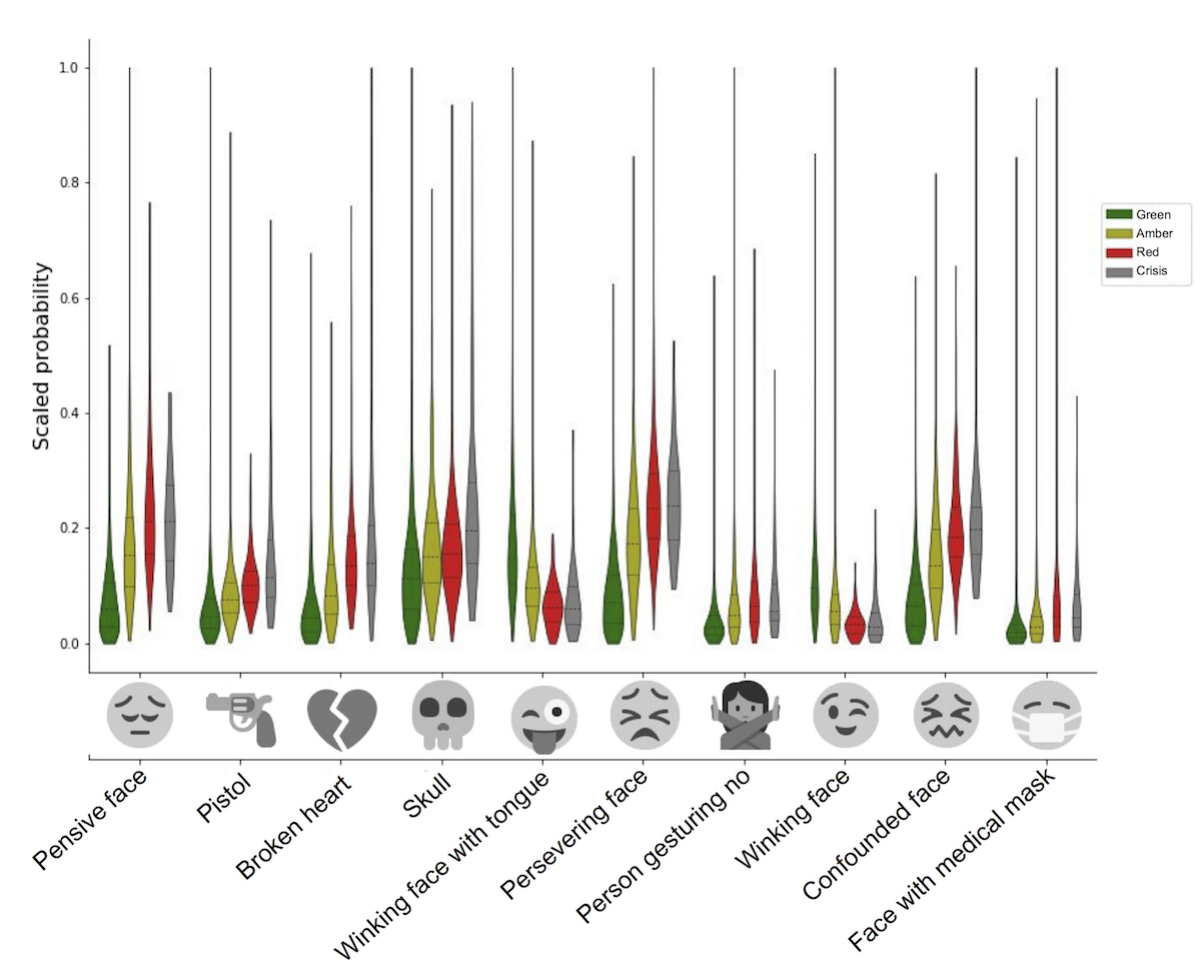
Violin plot showing the distributions of the 10 most discriminative emoji features across labeled classes. The classes are according to label with crisis in gray. The y-axis is the predicted scores for each emoji that have been scaled to the 0-1 interval. The emojis across the y-axis are marked with their images and their official Unicode text labels. The emojis are ranked from the most to least important feature (left to right).

In Figure 3, we show the distribution of the mean emoji features for the top 10 most important features when using the mean emoji feature across sentences (64 total features). We noted that the interpretation and even visual representation of these emojis vary greatly, and these emojis were not used in the social media posts but were extracted by DeepMoji [38]. For example, the pistol emoji has been replaced by a ray gun or water gun in most platforms. From these distributions, it is clear that there is considerable variability across posts. This visualization also highlights the difficulty in discriminating the varying levels of risk when compared with the no risk posts. Of these top 10, 2 winking emojis are negatively correlated with risk, marking the importance of a positive sentiment. As expected, the negative emojis are more important, with the pistol, skull, and broken heart emoji ranked in the top 5.

**Figure 4:**
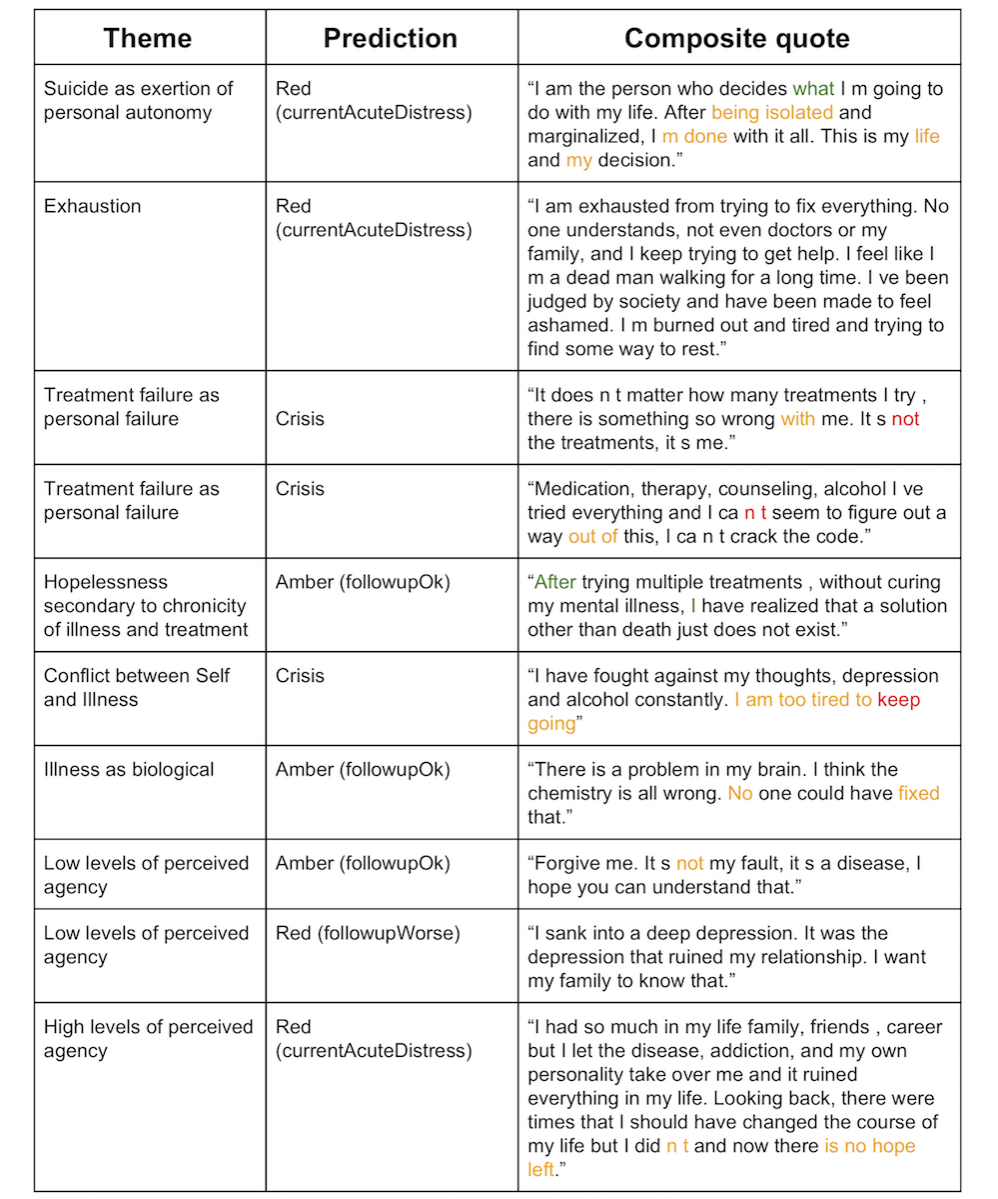
Predictions and highlights of suicide-related composite quotes from Furqan and colleagues. Words that changed predictions are color coded. Replacing a yellow or red word with an unknown word shifts the prediction to a less severe class by 1 or 2 levels, respectively, (ie, replacing a yellow word in text that is classified as crisis would change the prediction to red while a red word would change it to amber). In contrast, replacement of green words will result in more severe predictions.

To better understand judgments made by our trained classifier, we present predictions in Figure 4 on a set of composite quotes and their themes from a study of suicide notes [34]. For each quote, we presented the initial prediction (with the granular/fine-grained prediction in parentheses). Across the 10 quotes, 3 were classified as crisis, 4 as red, and 3 as amber. One of the amber classifications is under the “Hopelessness secondary to chronicity of illness and treatment” theme, further suggesting that our system may not recognize expressions of hopelessness.

All words were iteratively masked to indicate their effects on the predicted class (see Methods section). In Figure 4, words that affected predictions are color coded. The colored words are important for indicating severity as removing them makes the quotes appear less severe to our system. Examining these words suggests that negations affected severity (eg, “not,” “can’t”). In the quotes, negations seemed to indicate a perceived failure or not having done or achieved something the person felt they ought to. Expressions of hopelessness (ie, “no hope left”) were also important in classifying quotes as severe by our system. Words reflecting an unwillingness or inability to continue were also important (ie, “I’m done,” “I am too tired to”) as were words indicating loneliness (ie, “being isolated”). In contrast, replacing a green word with an unknown word shifted the predicted class to a more severe category (eg, from *red* to *crisis*). On examining the nature of the green words (ie, “what,” “after”), it was not clear why these words were important for lessening the severity of the quotes.

For 2 of the quotes predicted as red, no words were highlighted, suggesting that, in these instances, many words were key to the prediction. Overall, the quotes would all be flagged as requiring some level of moderator attention, and for the most part, the nature of words that were important in classifying the severity of quotes made conceptual sense.

## Discussion

We have shown that there are highly informative signals in the text body alone of posts from the Reachout.com forum. More specifically, we identified a transfer learning approach as particularly useful for extracting features from raw social media text. In combination with the training of classifiers using AutoML methods, we showed that these representations of the post content can improve triage performance without considering the context or metadata of the posts. These methods take advantage of the large amount of unlabeled free text that is often available to diminish the need for labeled examples. We also showed that these methods can generalize to new users on a support forum, for which there would not be preceding posts to provide context on their mental states. By combining the pretrained language models with AutoML, we were able to achieve state-of-the-art macro-F1 on the CLPsych 2017 shared task. Our content-only approach could be complemented by previous work, which used hand-engineered features to account for contextual information, such as a user’s post history or the thread context of posts [26,47]. Future developments could also include multiple types of media (eg, text, photos, videos) that are often present on social media to better assess the subtleties of users’ interactions [48].

Our current approach follows methods outlined by Radford et al [20] to fine-tune the language model that was previously pretrained on a large corpus of books. This fine-tuning step allows the model to learn the characteristics of the Reachout.com text. We show that increasing the amounts of in-domain unlabeled data for fine-tuning improves classification performance and has yet to reach a plateau. Further work will be instrumental in defining when and how to fine-tune pretrained language models better [49]. For tasks with limited data availability, the ability to adapt and fine-tune a model on multiple intermediate tasks could be a particularly worthwhile approach, as demonstrated by the Universal Sentence Encoder and others [39,50]. However, it is unclear how these large language models can retain and accumulate knowledge across tasks and datasets. Notably, it has been reported that these large pretrained language models are difficult to fine-tune and that many random restarts may be required to achieve optimal performance [51,52].

We compared the use of AutoML tools, such as Auto-Sklearn and TPOT, to generate classification pipelines with a variety of features extracted from free text. We also identified them as sources of variability in the final scores of our system. When developing our top-performing systems with features extracted from a fine-tuned GPT and using Auto-Sklearn on 20 trials, we obtained macroaverage F1 scores ranging from 0.6156 to 0.4594. In part, this is because of the small size of the dataset and the weighted focus of the macroaverage F1 metric toward the *crisis* class with relatively fewer instances. Further experiments, although computationally intensive, could help distinguish the amount of variability that is inherent in the language model fine-tuning process.

There are a variety of limitations, depending on the use of the approaches we benchmarked. Further experiments would be needed to determine if Reachout.com moderator responsiveness improves when more accurate classifiers are used. The present system performance cannot be extrapolated too far into the future because of changes in the population of users on the forum, shifting topics discussed or variations in language used. Furthermore, it is important to note that any implemented system would require ongoing performance monitoring.

To further understand how our trained models would perform in a new context, we assessed performance on an independently collected dataset and composite quotes that were derived from suicide notes. All composite quotes were flagged as requiring moderator attention. Our classifiers generalize to some degree on the UMD Reddit Suicidality Dataset, which approximates the task outlined for Reachout.com. We noted that the Reddit user base is not specific to Australia, is not targeted explicitly to youth, and may have substantially different topics of discussion than Reachout.com. This performance is primarily driven by good accuracy on the *no risk* or *green* class. We observed that the features derived from the fine-tuned GPT model perform worse than those from the default GPT model, indicating that this model might be specific to unique features of Reachout.com. Future studies could determine whether multiple rounds of fine-tuning on different datasets increase accuracy.

We manually reviewed the errors made by the best-performing system (Auto-Sklearn classifier with the GPT fine-tuned features). The most worrisome prediction errors occur when the classifier mistakes a crisis post for one of lesser importance, which could potentially delay a moderator response. When posts were not classified as crisis posts (but should have been), this was often due to vague language referring to self-harm or suicide (eg, “time’s up,” “get something/do it,” “to end it,” “making the pain worse”). Sometimes, forum users deliberately referred to self-harm or suicide with nonstandard variations, such as “SH” or “X” (eg, “attempt X,” “do X”). Future work could be instructive in determining whether these words are associated with higher levels of distress/crisis relative to the words they are meant to replace. Alternatively, custom lexicons might be developed to capture instances of self-harm or suicide represented by vague language or nonstandard variations.

In some failure cases (ie, posts that should be classified as being of higher risk than they were), the classifier did not notice expressions of hopelessness, which may cue the imminence of risk. Other prominent failure cases were instances when the classifier did not notice a poster’s dissatisfaction with mental health services that provide real-time help (eg, suicide call-back services and crisis helplines, etc). According to the labeling scheme, these posts should be classified as red. However, this dissatisfaction was often conveyed in diverse and highly contextualized ways, likely making it difficult for the system to identify. There were also posts that did not indicate imminent risk but described sensitive topics such as feeling lonely or losing a parent. These were often misclassified as green (when they should have been amber), possibly because they also contained positive language, or the sensitivity of the topic was difficult for the system to grasp.

In some of these failure cases, it may have been useful to take into account the previous post; eg, when the post in question is short or vague, the system may classify the level of risk more accurately if the previous post expresses a high level of concern about the poster or tries to convince the poster to seek immediate help.

Neural networks can build complex representations of their input features, and it can be difficult to interpret how these representations are used in the classification process. In a deeper analysis of DeepMoji features, we identified the most important emoji for classification and found that the emotional features follow a linear arrangement of expression at the class level corresponding to label severity. We also used input masking to iteratively highlight the contributions of individual words to the final classification. Such highlighting and pictorial/emoji visualizations could speed moderator review of posts. Ultimately, we believe the further development of methods to improve model interpretability will be essential in facilitating the work of mental health professionals in Web-based contexts.

In conclusion, we showed that transfer learning combined with AutoML provides state-of-the-art performance on the CLPsych 2017 triage task. Specifically, we found that an AutoML classifier trained on features from a fine-tuned GPT language model was the most accurate. We suggest this automated transfer learning approach as the first step to those building natural language processing systems for mental health because of the ease of implementation. Although such systems lack interpretability, we showed that emoji-based visualizations and masking can aid explainability.

## Appendix

Multimedia Appendix 1 Table including fine-grained annotations of Reachout posts.

## Abbreviations

AutoML: automated machine learning
CAMH: Centre for Addiction and Mental Health
CLPsych: Computational Linguistics and Clinical Psychology
GPT: Generative Pretrained Transformer
LIWC: Linguistic Inquiry and Word Count
TPOT: Tree-based Optimization Tool
VADER: Valence Aware Dictionary and sEntiment Reasoner
